# Epidural analgesia for a successful vaginal delivery in a parturient with Jarcho-Levin syndrome: case report

**DOI:** 10.1186/s12871-025-02978-3

**Published:** 2025-04-05

**Authors:** Eunah Song, Erik Romanelli

**Affiliations:** 1 Albert Einstein College of Medicine, 1300 Morris Park Avenue, Bronx, NY 10461 USA; 2 Albert Einstein College of Medicine Attending Anesthesiologist, Montefiore Medical Center, 111 E. 210th Street - Bronx, Bronx, 10467 NY USA

**Keywords:** Jarcho- Levin syndrome, Obstetric anesthesia, Epidural, Vaginal delivery, Case report, Spondylocostal dysostosis

## Abstract

**Background:**

There is a paucity of literature regarding obstetric anesthesia management in Jarcho-Levin Syndrome (JLS) patients and existing case reports are limited to pre-planned cesarean deliveries.

**Case presentation:**

The patient is a 21-year-old woman with JLS, spondylocostal dysostosis (SCD) subtype, who went through a full-term vaginal delivery with labor neuraxial analgesia. This case report details a successful trial of labor and delivery, which was permitted after an extensive testing of her baseline functional status, as well as a review of the difficulties present in the provision of both neuraxial and general anesthesia. There was an emphasis on multidisciplinary collaboration and planning throughout the patient’s peripartum course.

**Conclusion:**

A parturient with JLS was able to undergo full-term vaginal delivery using neuraxial analgesia and did not require general anesthesia or a cesarean section. Further research will enhance the management of obstetric anesthesia for patients with JLS.

## Background

Jarcho-Levin Syndrome (JLS) is a rare genetic disorder that manifests with distinct vertebral and thoracic abnormalities [1]. The incidence of JLS is estimated at 1:40,000 births worldwide [2]. Patients usually present with a short neck and trunk, resulting in a smaller stature [1]. Although JLS has been used as an umbrella term for two separate diseases, spondylothoracic dysplasia and spondylocostal dysostosis (SCD) are now acknowledged as two different entities [3]. Patients with SCD can have various rib deformities, including fusion, overgrowth, and complete absence of ribs [3]. The decreased size of their thoracic cavity due to these malformations gives rise to anesthetic challenges typical of patients with restrictive lung disease, which may complicate overall airway management.

Research on neuraxial anesthesia in patients with significant spinal deformities document several challenges. A review focusing on parturients with scoliosis reported patchy and asymmetric blocks in patients with surgically corrected scoliosis [4]. Positioning patients with previous spinal fusion surgeries is another concern, as the stiff rods make it difficult for patients to flex their spines [5]. Although placing neuraxial anesthesia in patients with scoliosis may require additional attempts, the success rate is high [4]. There are several recommendations regarding neuraxial anesthesia in patients with spinal deformities. Although no randomized trial exists to indicate the best neuraxial technique between an epidural, combined spinal-epidural (CSE), and a single spinal shot, current literature suggests that a CSE may be more reliable in regards to placement and spread of the anesthetic [4, 5]. Additionally, the use of a pre-puncture ultrasound may provide advantages like better anatomic identification and more information on the estimated depth and trajectory of the epidural [6].

The reports discussing JLS from the obstetric anesthesiologist’s perspective have been limited to planned cesarean deliveries with general anesthesia, using methods such as an awake fiberoptic intubation [7] or an awake video laryngoscopy-assisted tracheal intubation [8]; only one case was managed with a spinal anesthesia [9]. A more recent case report describes a parturient with SCD who received a modified rapid sequence induction of general anesthesia and transabdominal plane blocks for cesarean delivery [10]. Our case report discusses a parturient with JLS who underwent spontaneous vaginal delivery with an epidural block.

## Case presentation

Our patient is a 21-year-old G1P0 female with genetic testing confirmatory of SCD. Both parents were found to be carriers. She arrived at the obstetric service at 31-weeks gestation, citing difficulty in obtaining prenatal care due to her recent arrival to the U.S. and the COVID-19 pandemic. She was born without a shoulder blade and six missing ribs on her left side. In childhood, she had two spinal surgeries including a cervical spine fusion and an attempted correction of kyphoscoliosis at the ages of 1 and 13, respectively.

**Fig. 1:**
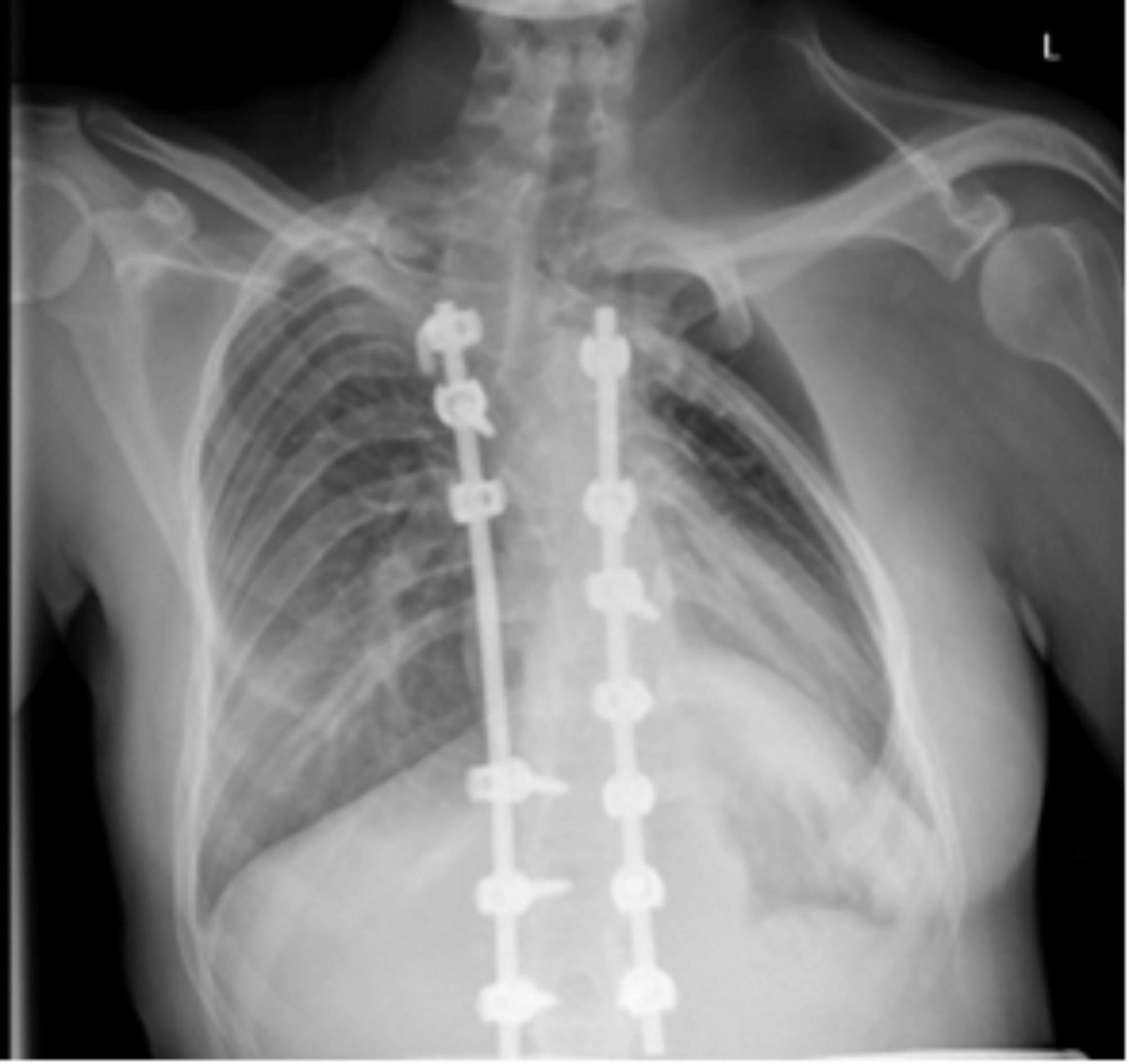
Chest x-ray showing displacement of the left scapula and diaphragm

The obstetrics team arranged for consultations with pulmonology and anesthesiology. A new chest x-ray revealed a displaced left scapula and diaphragm, along with findings consistent with left lung hypoplasia (Fig. 1). The patient had a baseline oxygen saturation of 94% and remained at this saturation throughout her third trimester office visits. She reported a history of recurrent pulmonary infections, with her most recent hospitalization for pneumonia occurring at 7-weeks gestation, but denied any active infections since then. The patient’s spirometry results were consistent with a severely restricted lung disease pattern with FEV1 28% of predicted value, FVC 29% of predicted value, and FEV1/FVC 98% of predicted value. The results of the echocardiogram were grossly normal, with an ejection fraction of 60%, minimal mitral and tricuspid valve regurgitation, and no evidence of right-heart strain.

During the anesthesiology consultation, it was noted that the patient had spinal deformities with disproportionate shoulders and a severely limited range of motion around the neck. Examination of the lumbar region revealed a surgical scar that extended downwards to the L1-L2 level. The patient reported having chronic back pain pre-dating the pregnancy and that she had been taking oxycodone/acetaminophen (Percocet) for the past several years. Her airway examination was notable for a Mallampati-2 score, > 3 finger-breadths thyromental distance, and adequate mouth opening. The patient’s height was 155 cm and her body mass index was 24.6 kg/m^2^. There were no anesthetic records available from her previous spine surgeries discussing airway management.

**Fig. 2:**
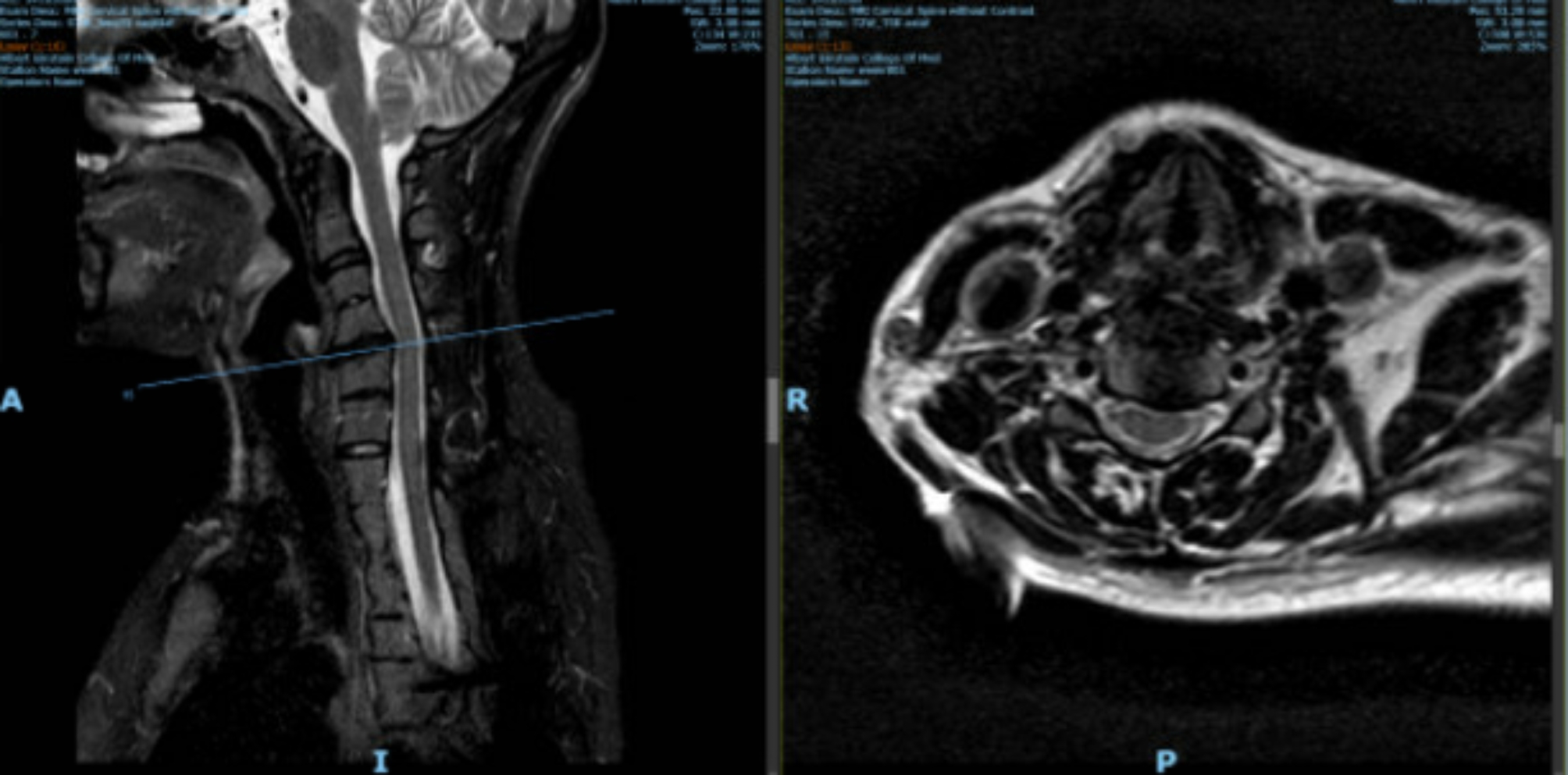
MRI of the cervical spine was significant for multilevel degenerative changes

**Fig. 3:**
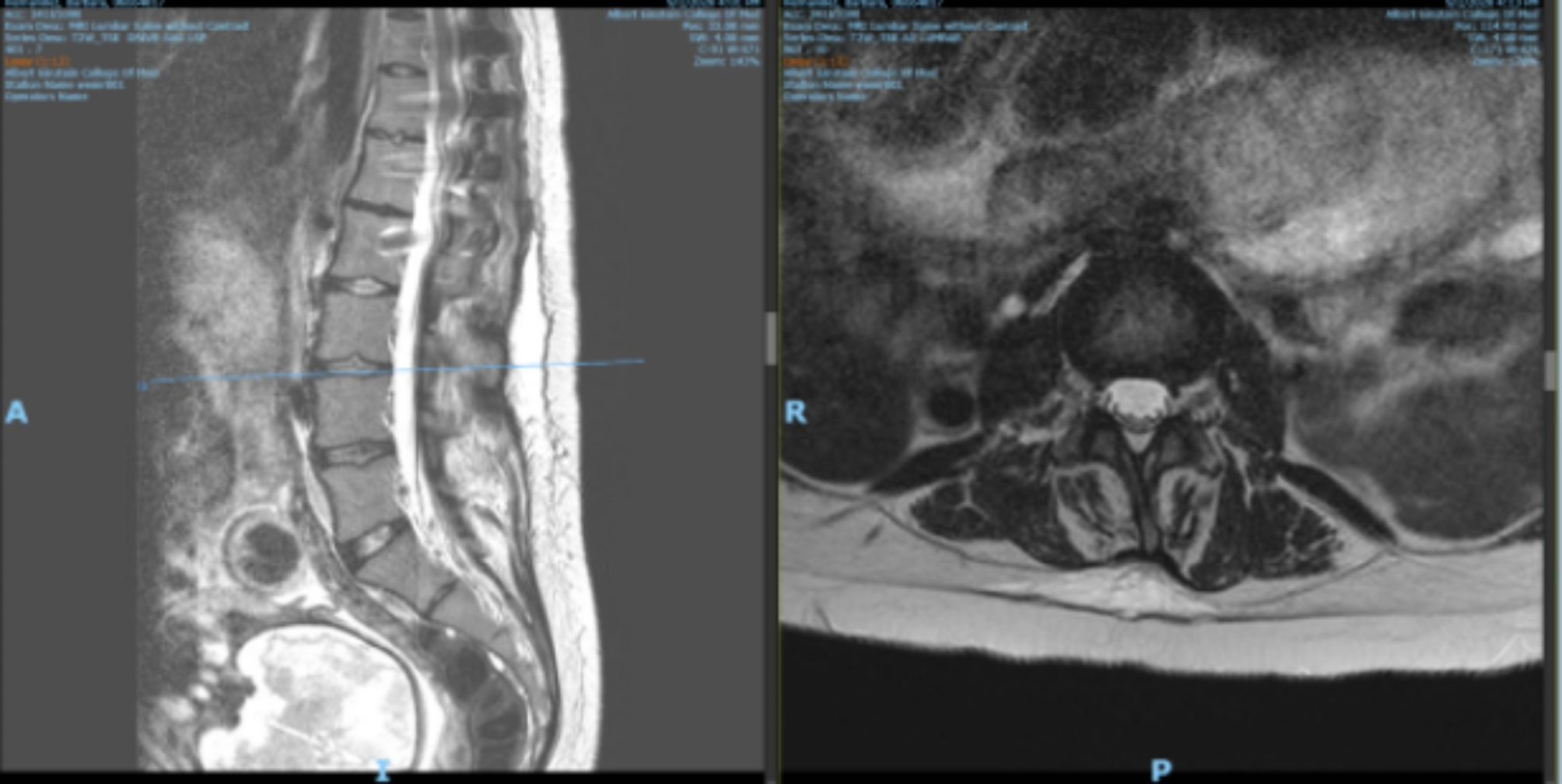
MRI of the lumbar spine confirmed the presence of surgical rods

The patient went into latent labor at 37-weeks gestation, presenting with minimal cervical change, irregular contractions, and intact membranes. She was not able to obtain her outpatient MRI in time. At the time of presentation, the patient denied any respiratory complaints. A focused cervical and lumbar MRI (excluding the thoracic region for expediency) was obtained while the patient was still in latent labor to assure that there were no findings that might preclude neuraxial analgesia for labor. MRI of the cervical spine (Fig. 2) was significant for vertebral body fusion from C7-T6 and multilevel degenerative changes. A disc osteophyte was identified at the C4-C5 level causing mild spinal canal stenosis and indentation of the spinal cord. MRI of the lumbar spine (Fig. 3) confirmed the presence of surgical rods extending to L2. It was otherwise unremarkable, with no significant spinal canal or neural foraminal stenosis. The thecal sac ended at the level of the L5 vertebral body, and the conus medullaris ended near the L2–L3 interspace.

It was determined that the patient was at her baseline functional status therefore she could attempt a trial of labor, with the possibility of an assisted second stage if there was any change in her functional status. Difficult airway equipment, including a C-MAC fiberoptic scope, was prepared in a dedicated operating room. An ENT specialist was on standby in case a surgical airway was needed. The patient’s cervical exam indicated 4 cm of cervical dilation, 80% cervical effacement, and a score of -3 for fetal station. She experienced regular contractions every six minutes upon return from the MRI. The placenta was left-lateral and the fetal heart rate tracing was category I at this time. An amniotomy was performed and a fetal scalp electrode was placed for better fetal monitoring during the labor epidural placement.

The patient required additional positioning maneuvers to be seated for the epidural placement. Following recommendations for parturients with scoliosis, the doctors focused on maximally flexing the spine, to widen the interspinous spaces and align the iliac crests as effectively as possible. During standard epidural positioning, multiple adjustments were needed to minimize her pain and discomfort. The anesthesiologist first attempted a traditional midline approach for labor epidural anesthesia. A dural puncture with a Gertie-Marx spinal needle was attempted but there was no return of cerebrospinal fluid (CSF) following a loss of resistance to air injection. The anesthesiologist then attempted a paramedian approach, positioning the needle 1–2 cm lateral to the spinal process on the convex side of the spinal curvature. A 9 cm epidural Tuohy needle was re-positioned and another attempt was made but there was no return of CSF. At this time, the anesthesiologist decided to thread the catheter and carefully assess analgesia with a slow load of 10 ml of 0.25% bupivacaine. The needle insertion depth was 6 cm, the catheter type was multiport 19G, and the catheter at skin depth was 11 cm.

Within twenty minutes of administration of the epidural, the patient was insensate to ice to the level of T9 and bilateral analgesic block was achieved. Her analgesia scores were 9 out of 10 prior to the epidural and 1 out of 10 within 15 min of the loading dose. She did not require any epidural “top-offs” (additional doses of medication through the catheter). Epidural analgesia was maintained with an injection of fentanyl preservative-free 100MCG through the epidural and a continuous infusion of 0.0625% bupivacaine combined with 2 mcg of fentanyl per mL.

The patient progressed to full dilation within two hours without the need for oxytocin augmentation and had a successful spontaneous vaginal delivery after twenty minutes of pushing unassisted. The newborn male weighed 2.565 kg and had an Apgar score of 9 at one and five minutes. The umbilical vein pH was 7.26. Approximately 2.5 h after delivery, a post-anesthesia evaluation was performed. The patient was awake and alert with no anesthetic complications. She was hemodynamically stable and breathing spontaneously without the need for assisted ventilation. 9 h post-delivery, the patient was in a stable condition with no headache or neurological issues. She was ambulating with no deficits. The patient had an uneventful hospital course and was discharged on postpartum day 2.

## Discussion and conclusions

Our report is the first documented case of a successful full-term vaginal delivery with labor neuraxial analgesia in a patient with SCD. In terms of obstetric management, nearly all previously reported documented cases of JLS parturients [7–10] opted for a planned cesarean section after deeming that the maternal effort required during labor could potentially precipitate severe cardiorespiratory distress. Despite inherent concerns for airway management, concerns of neuraxial block inadequacy and/or failure have prompted the use of general anesthesia in JLS patients. It is certainly recognized that a history of corrective orthopedic surgery and/or hardware placement can complicate neuraxial procedures, as there is potential for epidural space obliteration, fibrosis, adhesions, and inflammation, which can cause inadequate spread of local anesthetics [11]. However, there are no absolute contraindications in attempting neuraxial analgesia for parturients with JLS.

Neuraxial anesthesia was preferred over general anesthesia in this case for several reasons. The patient’s severe restrictive lung disease secondary to lung hypoplasia posed a concern for respiratory complications. Furthermore, the patient reported experiencing hypoxia during her last surgery, an event consistent with research indicating that significant spinal deformities can increase the risk of hypoxia during anesthesia [12]. Additionally, the patient recalled being informed that she has sleep apnea. The American Society of Anesthesiologists expresses that patients with obstructive sleep apnea are more susceptible to significant postoperative respiratory compromise [13]. Given these risk factors, the care team thought it would be prudent to avoid general anesthesia. The COVID-19 pandemic also influenced the decision, as concerns of virus transmission via aerosolization had providers prefer neuraxial anesthesia over general anesthesia.

The patient had expressed her adamant desire to avoid cesarean delivery, if possible. Following antepartum assessments and expert consultations with pulmonology and cardiology, it was determined that a trial for vaginal delivery could be permitted in a Level IV regional perinatal health care center. A blood gas analysis was not performed before and during the labor because her pre-operative spirometry was consistent with her anatomic anomalies, she did not have any active respiratory complaints at the time of her presentation, and there was no obstruction noted despite her history of asthma. A baseline blood gas would have been considered and warranted had the case progressed to general anesthesia and there was concern regarding whether she could be safely extubated. For labor, it was not deemed necessary because the patient was at her baseline functional status.

The multidisciplinary planning between the anesthesiology, obstetrics, and pulmonology departments contributed to the successful outcome of this delivery. There was careful review of the potential complications that could occur during labor and the specialists discussed alternative interventions in a collaborative fashion. The patient was assessed 12 days prior to her induction date and encouraged to receive an early epidural placement, allowing for a “trial of labor analgesia”. However, if epidural analgesia was ineffective or vaginal delivery was not possible, the patient understood that alternative options, such as a cesarean section with neuraxial anesthesia (spinal, plain epidural, dural puncture epidural [DPE], CSE) or general anesthesia, were a real possibility. An integral component of interdisciplinary communication was the acknowledgment that there would be no true guarantee of successful conversion to surgical anesthesia if a cesarean delivery was necessary. Hence early discussions addressed lowering the threshold for conversion to a cesarean delivery if there were any signs of maternal intolerance or changes in the fetal tracing pattern. It was stressed upon all providers that an emergent situation should be avoided as much as possible since securing the airway under such circumstances would be less than ideal. The absence of the patient’s left shoulder blade and six ribs would pose difficulties in positioning the patient for any type of anesthesia. Furthermore, her cervical fusion causing severely limited neck extension, would complicate all intubations and airway management.

Prior to the administration of anesthesia, there were two unsuccessful attempts at a lumbar puncture before the anesthesiologist decided to proceed with a plain epidural. The original plan was a CSE or DPE, with placement of an epidural catheter. During the initial attempt, the anesthesiologist tried the traditional midline approach for the labor epidural; in the second attempt, they converted towards a modified paramedian approach. In both attempts, there was no return of CSF that would confirm that the needle was in the subarachnoid space. Due to the difficulty of positioning the patient and placing the catheter, more than thirty minutes had passed. At this point, the anesthesiologist prioritized adequate analgesia and proceeded to thread the catheter in the epidural space. After the epidural placement and initial load, the block level increased synchronously on both sides and analgesia was evenly distributed. Intensive dermatomal level checks were done using ice. The anesthesiologist was reassured that an adequate analgesic level had been achieved and did not attempt to reposition the catheter.

During this case, the delivery proceeded smoothly with no complications; however, several measures can be implemented to optimize results. In future cases, physicians may consider a CSE or DPE over a standard epidural. CSE analgesia in labor shows a decreased rate of catheter replacement in comparison to traditional epidurals [14] and they are also more reliable, as shown by the reduced incidences of breakthrough pain [15]. DPEs are growing in popularity and several studies have explored whether a DPE is comparable to a standard epidural in the setting of labor and delivery. While a systematic review of randomized controlled trials (RCT) comparing DPEs with epidural analgesia concluded insufficient evidence regarding both the benefits and risks of DPE [16], a more recent RCT reported results suggesting that DPEs result in faster onset of anesthesia and improved block quality in elective cesarean deliveries [17]. With the ongoing research, it is recommended that physicians remain updated to ensure the best clinical practices. In case of an unsuccessful epidural placement, anesthesiologists should consider threading an intrathecal catheter for labor analgesia instead of repositioning the epidural. Intrathecal catheterization offers multiple advantages such as rapid initiation of analgesia and avoidance of repeated accidental dural punctures (ADP) [18]. Although there are some associated complications such as spinal cord damage, drug administration errors, and infection, studies have shown that an intrathecal catheter insertion after an ADP can provide effective labor analgesia and cesarean section anesthesia for parturients [18].

Lastly, having an anesthesiologist proficient in neuraxial ultrasound can improve the accuracy of the epidural placement. Preprocedural neuraxial ultrasound increases the precision and success of neuraxial anesthesia by identifying lumbar intervertebral spaces more accurately and providing information on the depth of the epidural space [19]. During this case, a neuraxial ultrasound of the back was performed during the patient’s initial consult with anesthesiology to determine that an epidural space did exist and that it had not been obliterated during her prior back surgeries. It was not used during the actual neuraxial placement but we recommend its use for better midline identification.

Parturients with JLS present a challenge for obstetric anesthetic management due to the potential difficulties of both neuraxial and general anesthesia. Whereas prior case reports have primarily described cesarean section deliveries performed under general anesthesia, this report presents the first documented case of a JLS patient who underwent a spontaneous vaginal delivery with adequate epidural labor analgesia. This is significant given that deliveries that do not require cesarean section or general anesthesia are generally associated with improved health outcomes for both the mother and neonate. Multidisciplinary planning ensures that there is a team of specialists familiar with the patient’s condition, who can provide expert guidance regarding the delivery and also be prepared for backup scenarios in the event of complications. Given the paucity of information on parturients with JLS, particularly within the context of neuraxial blocks and vaginal delivery, we hope that this case report will serve as a valuable resource for other physicians.

## Data Availability

No datasets were generated or analysed during the current study.

## Abbreviations

JLS: Jarcho-Levin Syndrome
SCD: Spondylocostal dysostosis
CSE: Combined spinal-epidural
CSF: Cerebrospinal fluid
DPE: Dural puncture epidural
RCT: Randomized controlled trial
ADP: Accidental dural puncture
